# Distinct Gene Profiles of Bone Marrow-Derived Macrophages and Microglia During Neurotropic Coronavirus-Induced Demyelination

**DOI:** 10.3389/fimmu.2018.01325

**Published:** 2018-06-11

**Authors:** Carine Savarin, Ranjan Dutta, Cornelia C. Bergmann

**Affiliations:** Department of Neurosciences, NC-30, Lerner Research Institute, Cleveland Clinic Foundation, Cleveland, OH, United States

**Keywords:** macrophages, microglia, central nervous system, demyelination, viral encephalomyelitis

## Abstract

Multiple Sclerosis (MS) is a chronic inflammatory disease of the central nervous system (CNS) characterized by demyelination and axonal loss. Demyelinating lesions are associated with infiltrating T lymphocytes, bone marrow-derived macrophages (BMDM), and activated resident microglia. Tissue damage is thought to be mediated by T cell produced cytokines and chemokines, which activate microglia and/or BMDM to both strip myelin and produce toxic factors, ultimately damaging axons and promoting disability. However, the relative contributions of BMDM and microglia to demyelinating pathology are unclear, as their identification in MS tissue is difficult due to similar morphology and indistinguishable surface markers when activated. The CD4 T cell-induced autoimmune murine model of MS, experimental autoimmune encephalitis (EAE), in which BMDM are essential for demyelination, has revealed pathogenic and repair-promoting phenotypes associated with BMDM and microglia, respectively. Using a murine model of demyelination induced by a gliatropic coronavirus, in which BMDM are redundant for demyelination, we herein characterize gene expression profiles of BMDM versus microglia associated with demyelination. While gene expression in CNS infiltrating BMDM was upregulated early following infection and subsequently sustained, microglia expressed a more dynamic gene profile with extensive mRNA upregulation coinciding with peak demyelination after viral control. This delayed microglia response comprised a highly pro-inflammatory and phagocytic profile. Furthermore, while BMDM exhibited a mixed phenotype of M1 and M2 markers, microglia repressed the vast majority of M2-markers. Overall, these data support a pro-inflammatory and pathogenic role of microglia temporally remote from viral control, whereas BMDM retained their gene expression profile independent of the changing environment. As demyelination is caused by multifactorial insults, our results highlight the plasticity of microglia in responding to distinct inflammatory settings, which may be relevant for MS pathogenesis.

## Introduction

Multiple Sclerosis (MS) is a chronic inflammatory disease of the central nervous system (CNS), characterized by demyelination and axonal damage. Active demyelinating lesions are characterized by CD8 T cells, CD4 T cells expressing both Th1 and Th17 cytokines, bone marrow-derived macrophages (BMDM) and activated CNS resident microglia (1, 2). Myeloid cells activated by T cell effector functions are thought to participate in tissue damage by removing or “stripping” myelin (3), and secreting toxic factors, such as reactive oxygen species, nitric oxide and the pro-inflammatory cytokines, tumor necrosis factor (TNF), and IL-1β (4, 5). Activated microglia also secrete chemokines, which recruit innate and adaptive immune cells into the parenchyma, further amplifying the destructive inflammatory response (5). However, both BMDM and microglia effector functions are highly heterogeneous depending on the environment and may not only contribute to disease progression but also to resolution (6, 7). For example, by removing apoptotic cells and debris, their phagocytic activity favors tissue repair and is essential for disease resolution (3). In addition, both cell populations secrete anti-inflammatory cytokines, such as IL-10 and TGF-β, as well as trophic factors, which provide an environment that promotes tissue repair and neuronal protection (8). The heterogeneity of the inflammatory response associated with MS lesions at the cellular and functional levels, thus makes it difficult to establish detrimental versus disease resolving functions of BMDM and microglia in MS pathogenesis. In addition to the inherent limitations associated with sampling CNS tissues for longitudinal studies, the individual role of BMDM versus microglia as pathological mediators remains ambiguous due to morphological similarities and lack of reagents uniquely identifying each population. However, increasing evidence from animal models supports the concept that microglia and BMDM comprise two effector populations with distinct origins (derived from progenitors in the embryonic yolk sac and circulating monocytes respectively) and functions during MS and other neuroinflammatory disorders (9).

A variety of murine models, including autoimmune- and viral-induced demyelination, have been developed to study pathogenic features of MS (10). The most common is the experimental autoimmune encephalomyelitis (EAE), an autoreactive CD4 T cell-induced autoimmune demyelination characterized by infiltration of myelin-specific Th1 and Th17 cells, BMDM and microglial activation (11, 12). Pathogenesis during EAE is associated with temporally distinct microglial activation and BMDM CNS infiltration. Early microglia activation is insufficient to trigger clinical disease, whereas delayed CNS recruitment of BMDM directly correlates with disease progression. Importantly, depletion of BMDM but not microglia inhibits EAE (13, 14). Similarly, mice deficient in CCL2 (CCL2^−/−^), a chemokine essential for inflammatory monocyte recruitment into the CNS (15), are resistant to EAE (16). In support of detrimental BMDM functions, a combined histological and gene profiling study showed that demyelination is mediated by BMDM associated with nodes of Ranvier, whereas debris clearance is achieved by microglia (17). Altogether, studies in the EAE model demonstrate that BMDM recruitment into the CNS is essential for the process of myelin loss and clinical manifestation.

Inflammatory demyelination is also induced following infection with two natural viral mouse pathogens, Theiler’s murine encephalomyelitis virus (TMEV) and members of the neurotropic mouse hepatitis viruses (MHV). TMEV infection induces an autoimmune disease in which BMDM are essential for both viral persistence and demyelination (18, 19). However, the function of BMDM as a main reservoir of active viral replication during chronic TMEV infection, limits efforts to assess their role in demyelination independent of virus load (20). In contrast, infection with the non-lethal glia tropic MHV strain designated JHMV predominantly targets oligodendrocytes (OLG) and to a lesser extent microglia and astrocytes. Viral replication peaks at day 5 post infection (p.i.), but infectious virus is reduced below detection by day 14 p.i. Acute infection initiates rapid CNS recruitment of predominantly BMDM, but also neutrophils and NK cells, followed by infiltration of both CD8 and CD4 T cells, as observed in active MS lesions. The T cell response, which is essential to reduce viral replication, is highly Th1 polarized with no evidence of IL-17 or GM-CSF production (21–23). Importantly, T cell-mediated virus control coincides with initiation of demyelination, which peaks between days 14–21 p.i. after infectious virus is cleared (24, 25). Although OLG tropism is a requirement for demyelination, immunodeficient mice demonstrated that infection of OLG in the absence of adaptive immunity is insufficient to cause demyelination. However, transfer of either virus-specific CD4 or CD8 T cells into virus infected immunodeficient mice leads to demyelination (26, 27). Furthermore, IFN-γ dependent control of infectious virus within OLG and no evidence for OLG apoptosis, suggested that direct T cell-mediated cytolysis of OLG does not play a major role in myelin loss (28). This implicates T cell activated BMDM and microglia as the most probable mediators of myelin destruction. Moreover, both myeloid populations are abundant in lesions and occasionally associated with damaged axons (29). However, in contrast to EAE, genetic or chemical depletion of monocytes during JHMV infection does not alter disease severity, virus replication or myelin loss (30, 31), suggesting that BMDM are dispensable for JHMV-induced demyelination.

This study takes advantage of the distinct tissue environments established during EAE and JHMV infection to characterize temporal alterations in gene expression profiles of BMDM versus microglia in a Th1 biased demyelination model. To date, we are not aware of any reports evaluating the signature profile of microglia associated with pathogenic functions during demyelination. The results reveal that CNS infiltrating BMDM rapidly establish a characteristic profile including M1 and M2 markers, which prevails throughout infection as the population declines. By contrast, gene expression in microglia is only prominently altered remote from viral control concomitant with demyelination; distinct from BMDM, the gene expression pattern is skewed to a highly pro-inflammatory and phagocytic profile. The results overall highlight the plasticity of microglia responses in distinct inflammatory settings, which may be relevant for MS pathogenesis at distinct stages of disease.

## Materials and Methods

### Mice

Wild-type (WT) C57BL/6 mice were purchased from the National Cancer Institute (Frederick, MD, USA). Homozygous CCL2 deficient (CCL2^−/−^) mice were originally obtained from B. J. Rollins (Dana-Farber Cancer Institute, Boston, MA, USA). CX3CR1^GFP/GFP^ (B6.129P-*Cx3cr1^tm1Litt^*/J) and CCR2^RFP/RFP^ (B6.129(Cg)-*Ccr2^tm2.1Ifc^*/J) mice were purchased from the Jackson Laboratory (Bar Harbor, ME, USA) and crossed to generate CX3CR1^GFP/+^CCR2^RFP/+^ mice. Transgenic mice were bred and maintained at the Biological Research Institute under sterile conditions. All procedures were preformed in compliance with the Cleveland Clinic Institutional Animal Care and Use Committee approved protocols.

### Virus and Infections

The glia tropic JHMV neutralizing monoclonal antibody (mAb)-derived 2.2v-1 variant was used for all infections (32). Mice of both sexes between 6 and 7 weeks of age were infected in the left hemisphere with 1,000 PFU of JHMV diluted in endotoxin-free Dulbecco’s phosphate-buffered saline (PBS) in a final volume of 30 µl. Mice were monitored daily for clinical disease severity according to the following scale: 0, healthy; 1, hunched back and ruffled fur; 2, partial hind limb paralysis or inability to maintain the upright position; 3, complete hind limb paralysis; 4, moribund or dead.

### Isolation of CNS Mononuclear Cells, Flow Cytometry, and Cell Sorting

For analytical flow cytometry, anesthetized mice were perfused with ice-cold PBS, and resected brains and spinal cords homogenized using a Ten-Broeck tissue grinder as described (33). Tissue homogenates were adjusted to 30% percoll (Pharmacia, Uppsala, Sweden) and underlaid with 1 ml 70% percoll prior to centrifugation at 850 *g* for 30 min at 4°C. CNS mononuclear cell were recovered from the 30/70% interface, washed and resuspended in FACS buffer (PBS + 1% bovine serum albumin). Cells were blocked with anti-mouse CD16/CD32 (clone 2.4G2) mAb for 15 min on ice prior to staining. Staining was performed for 30 min on ice using fluorescein isothiocyanate, phycoerythrin, peridin chlorophyll protein complex (PerCP), or allophycocyanin (APC) conjugated mAb (all from BD Biosciences except where indicated) specific for CD45 (clone Ly-5), CD11b (clone M1/70), F4/80 (Serotec, Raleigh, NC, USA) and major histocompatibility complex (MHC) class II (clone 2G9). Cells were then washed twice in FACS buffer prior to analysis using a BD Accuri flow cytometer (BD Biosciences) and FlowJo software (Tree Star Inc., Ashland, OR, USA).

For cell purification, spinal cords from PBS-perfused CX3CR1^GFP/+^CCR2^RFP/+^ mice were finely minced with a razor blade. Minced tissues were enzymatically digested in RPMI 1640 medium containing 10% fetal calf serum, 0.5% collagenase D (100 mg/ml) Roche, Basel, Switzerland and 1% DNase I (1 mg/ml) (Sigma Aldrich, St. Louis, MO, USA) for 40 min at 37°C. Collagenase was then inactivated by addition of 1% 0.1 M EDTA for 5 min at 37°C prior centrifugation at 400 *g* for 7 min at 4°C. Spinal cord-derived cells from seven mice were pooled and isolated using percoll gradients as described above and then stained with CD11b-PerCP and CD45-APC for 30 min on ice. Spinal cord-derived BMDM (CD45^hi^CD11b^+^CCR2^RFP+^) and microglia (CD45^low^CD11b^+^CX3CR1^GFP+^) were purified using a FACSAria cell sorter (BD Biosciences) and resuspended in Trizol. Yields from 7-pooled mice ranged between 5.4–20 × 10^5^ cells for BMDM and 0.5–1.2 × 10^5^ cells for microglia depending on the time p.i. Microglia from naïve mice were used to assess baseline expression, whereas circulating monocytes were used as controls for CNS infiltrated BMDM after infection. Monocytes were isolated from blood treated with Gey’s solution to lyse red blood cells prior to staining and cell sorting.

### Gene Expression Profiling Using nCounter Analysis

RNA was prepared by extraction with TRIzol reagent (Invitrogen, Carlsbad, CA, USA) and Direct-zol RNA mini prep (Zymo Research, Irvine, CA, USA) according to the manufacturer’s instructions. Gene expression profiles were analyzed using the nCounter mouse Myeloid Innate Immune panel comprising 754 targets representing all major myeloid cell types and generated according to the manufacturer’s protocol (NanoString Technologies, Seattle, WA, USA). The NanoString nCounter system directly captures and counts individual mRNA transcripts using a multiplexed measurement system thereby omitting cDNA based amplification (34). Analysis was performed using nSolver Analysis Software v3.0 and Ingenuity pathway analysis (Qiagen, Hilden, Germany). Venn diagrams from individual gene lists and protein-protein interaction networks were constructed using Genespring (Agilent, Inc.) and STRING software (http://www.string-db.org).

### Reverse Transcription and Real-Time PCR

To confirm validity of Nanostring nCounter analysis, a small set of selected genes were analyzed by real-time PCR (Figure S1 in Supplementary Material). Following RNA extraction as described above, first-strand cDNA was synthesized using reverse transcriptase (Invitrogen) with oligo-dT and random primers (Promega, Madison, WI, USA) as described (35). Gene expression analysis was performed using a 7500 Fast real-time PCR system (Applied Biosystems, Foster city, CA, USA), SYBR Green Master mix (Applied Biosystems) and the following primers: GAPDH, 5′-CATGGCCTTCCGTGTTCCTA-3′ (forward) and 5′-ATGCCTGCTTCACCACCTTCT-3′ (reverse); IL15, 5′-TGAGGCTGGCATTCATGTCTT-3′ (forward) and 5′-TCCAGTTGGCCTCTGTTTTAGG-3′ (reverse); IL1rn 5′-AGATAGACATGGTGCCTATTGACCTT-3′ (forward) and 5′-CATCTCCAGACTTGGCACAAGA-3′ (reverse) and Arg1 5′-TGGGTGGATGCTCACACTGA-3′ (forward) and 5′-CAGGTTGCCCATGCAGATT-3′ (reverse). Transcripts levels were normalized to the housekeeping gene GAPDH and converted to a linearized value using the following formula: 2^(C^_T_^GAPDH-C^_T_^gene)^ × 1,000, where C_T_ represents the threshold cycle value.

### Histological Analysis

Following PBS perfusion, spinal cords were fixed in 10% neutral buffered Formalin, embedded in paraffin and sections stained with Luxol Fast Blue as described to visualize demyelination (36). For analysis of Iba1^+^ cells spinal cords from ice-cold PBS-perfused mice were quickly embedded in OCT and kept at −80°C until 10 µm sections were prepared. Sections were fixed with paraformaldehyde for 20 min, treated with blocking solution for 30 min and then stained with rabbit anti-Iba1 mAb (Wako, Osaka, Japan) overnight at 4°C. Goat anti-rabbit secondary Ab (Invitrogen) was added for 1 h at room temperature and sections mounted with Vectashield mounting medium (Vector Laboratories, Burlingame, CA, USA). Sections were analyzed using a Leica TCS confocal microscope.

## Results

### Infiltrating Macrophages Are Dispensable to JHMV-Induced Demyelination

**Figure 1:**
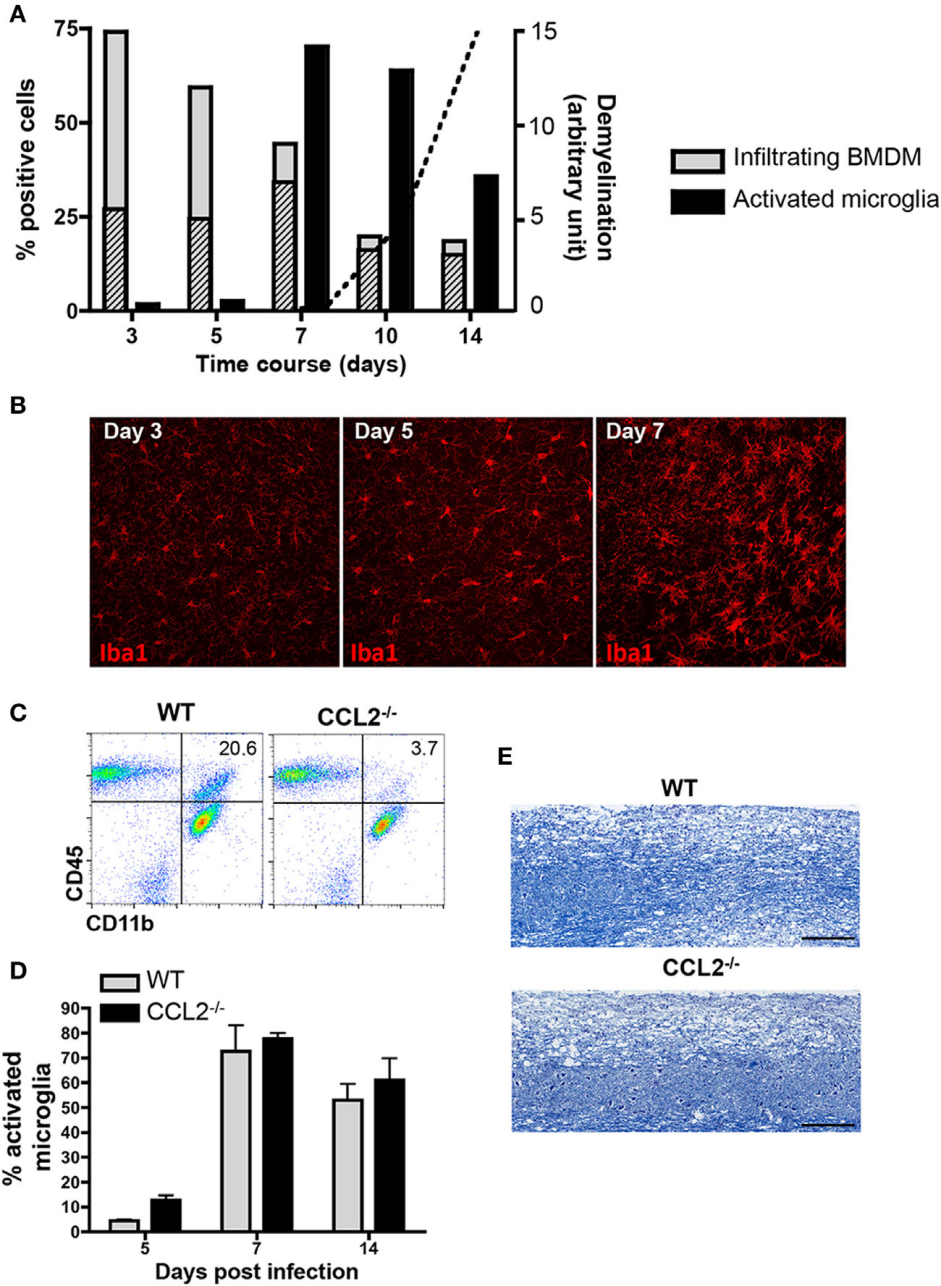
Demyelination correlates with microglia activation and is not affected in absence of bone marrow-derived macrophage (BMDM) central nervous system (CNS) infiltration. **(A)** Brains dissected from JHMV-infected wild-type (WT) mice were analyzed by flow cytometry for infiltrating CD11b^+^F4/80^+^ BMDM and activated CD45^low^CD11b^+^ microglia defined by their major histocompatibility complex (MHC) Class II^+^ phenotype between days 3 and 14 p.i. Gray bars depict the percentage of total CD11b^+^F4/80^+^ BMDM within infiltrating CD45^hi^ leukocytes, with striped bars representing the MHC Class II expressing BMDM fraction. Black bars represent the proportion of Class II^+^ cells within the microglia population. Data are from three-pooled mice per time point and representative of at least three experiments. The superimposed dotted line shows the relative kinetics and extent of demyelination in arbitrary units. **(B)** Microglia morphological changes at days 3, 5, and 7 p.i. were visualized by confocal microscopy of brain sections stained with anti-Iba1 monoclonal antibody (mAb). 40× magnification. Pictures are representative of four separate animals. **(C)** Brains of WT and CCL2^−/−^ infected mice at day 14 p.i. were analyzed for CD45 and CD11b expressing myeloid cells. Representative flow cytometry plots show a reduction of CD11b^+^ cells within the infiltrating CD45^hi^ population in the absence of CCL2. Numbers represent the percentage of CD11b^+^ cells within CD45^hi^ infiltrates. **(D)** Brains of infected WT and CCL2^−/−^ mice between days 5 and 14 p.i. were analyzed by flow cytometry for activated MHC Class II^+^ microglia Bar graphs depict the percentage of class II + cells within CD45^low^CD11b^+^ cells. Data represent the mean ± SEM of three experiments with *n* = 3-pooled mice per group per experiment. **(E)** Demyelinating lesions within spinal cords of WT and CCL2^−/−^ mice at day 21 p.i. were visualized by Luxol Fast Blue staining.

To better characterize reactivity of microglia and infiltrating BMDM following JHMV infection, we initially monitored CNS infiltration of BMDM, as well as upregulation of MHC class II as an activation marker on both CNS BMDM and microglia by flow cytometry. BMDM with a typical CD45^hi^CD11b^+^F4/80^+^ phenotype comprised the majority of inflammatory leukocytes as early as day 3 p.i. and then progressively decreased as virus replication is controlled by T cells. At the onset of demyelination at day 10 p.i., the BMDM population stabilized at ~10% of the infiltrating leukocytes (Figure 1A). BMDM initially infiltrated as MHC class II^lo^ expressing cells, but the vast majority upregulated MHC class II by day 7 p.i. MHC class II expression on microglia was sparse at days 3 and 5 p.i., but rapidly increased by day 7 p.i. and then gradually declined by day 14 p.i. (Figure 1A). These kinetics supported that microglia and BMDM activation peaks delayed relative to peak BMDM accumulation and coincides with peak T cell IFN-γ production (36, 37). Enhanced activation of microglia at day 7 p.i., compared to earlier times p.i., was also supported by progression of morphological changes, evidenced by enlarged cell bodies and retracted and thickened processes (Figure 1B). The decline of BMDM, but an ongoing activation phenotype of microglia at the time of evident demyelination implicated microglia as mediators of tissue damage during JHMV encephalomyelitis.

Biochemical depletion of peripheral monocytes indeed supported that BMDM are not essential to tissue destruction in JHMV-infected mice (30). Data from our own laboratory further demonstrated that the chemokine CCL2 is essential for BMDM accumulation within the CNS (31). The absence of CCL2 resulted in an ~80% reduction of BMDM at all time points, including day 14 p.i. (31) and (Figure 1C) when demyelination is prominently evident in WT mice. Nevertheless, microglia activation, as monitored by MHC class II expression, was independent of CCL2 (Figure 1D). Most importantly, the absence of CCL2-dependent BMDM within the CNS did not alter demyelination (Figure 1E). Similar myelin loss at day 21 p.i. comparing WT and CCL2^−/−^ infected mice supported the concept that microglia mediate demyelination during JHMV infection.

### Characterization of BMDM and Microglia Under Homeostatic Conditions

**Figure 2:**
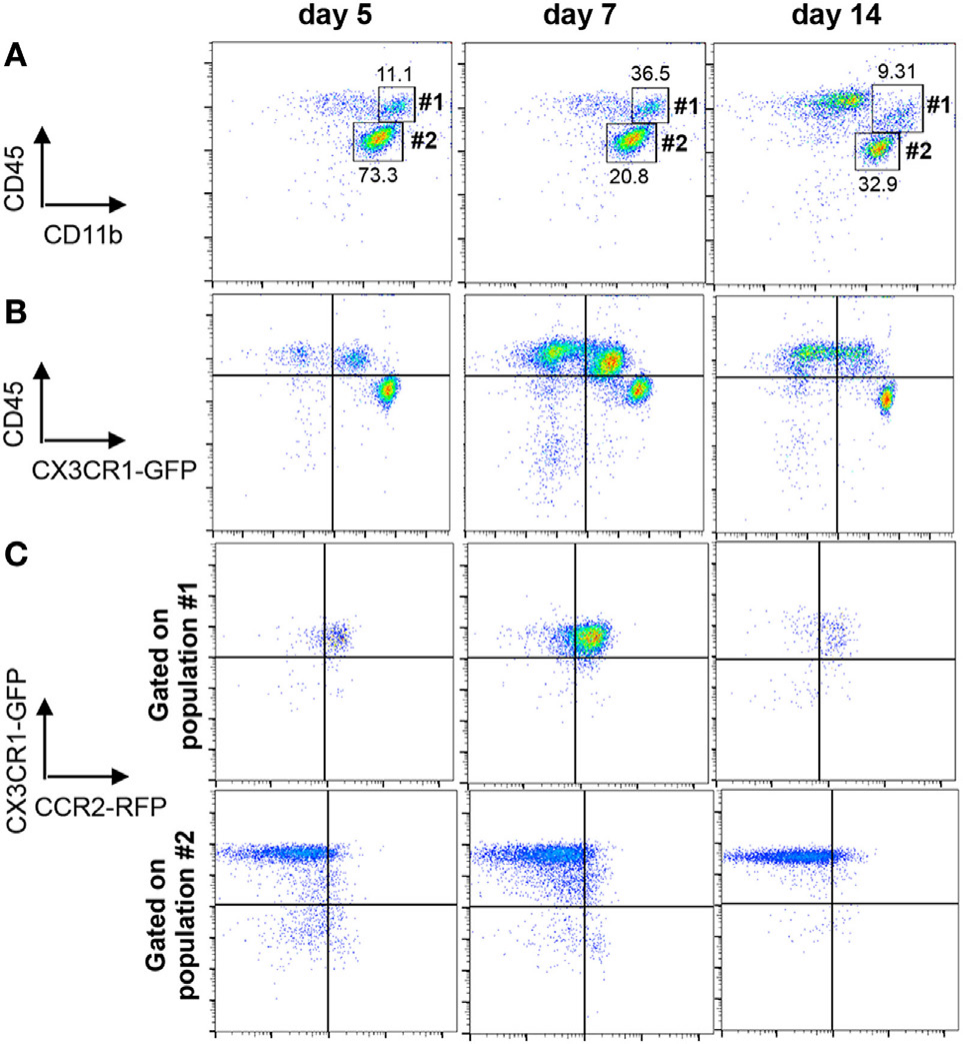
Distinction of microglia and infiltrating bone marrow-derived macrophage (BMDM) within the central nervous system (CNS) of CX3CR1^GFP/+^CCR2^RFP/+^ mice. **(A)** Representative flow cytometry plots of CD45^hi^CD11b^+^ BMDM (population #1) and CD45^low^CD11b^+^ microglia (population #2) gated on total CD45 cells from JHMV-infected spinal cords at the indicated time points. **(B)** Cells from Panel A were analyzed for differential CX3CR1^GFP^ expression on CD45^hi^ and CD45^low^ populations. **(C)** Cells from Panel A gated on CD45^hi^CD11b^+^ BMDM (population 1) or CD45^low^CD11b^+^ microglia (population #2) were assessed for CX3CR1^GFP^ and CCR2^RFP^ expression. All data are acquired from mechanically disrupted tissue of JHMV-infected CX3CR1^GFP/+^CCR2^RFP/+^ mice and representative of two separate experiments with at least three mice per time point per experiment.

We next evaluated effector functions of BMDM versus microglia associated with JHMV-induced demyelination by comparing gene expression profiles using nCounter analysis of mRNA isolated from purified BMDM and microglia of infected CX3CR1^GFP/+^CCR2^RFP/+^ mice. Characteristic expression of CX3CR1^GFP^ and CCR2^RFP^ on CD45^high^CD11b^+^ BMDM (population #1) and CD45^low^CD11b^+^ microglia (population #2) is shown in Figure 2 throughout days 5–14 p.i. Microglia were characterized by high expression of CX3CR1 and undetectable CCR2 expression (Figures 2B,C) similar to other inflammatory models (17, 38). In contrast, CNS infiltrating BMDM expressed CCR2 and low levels of CX3CR1 compared to microglia (Figure 2B). Co-expression of CCR2 and CX3CR1 was maintained on BMDM at all time points p.i. and no CX3CR1^−^ cells were detectable (Figure 2C).

**Figure 3:**
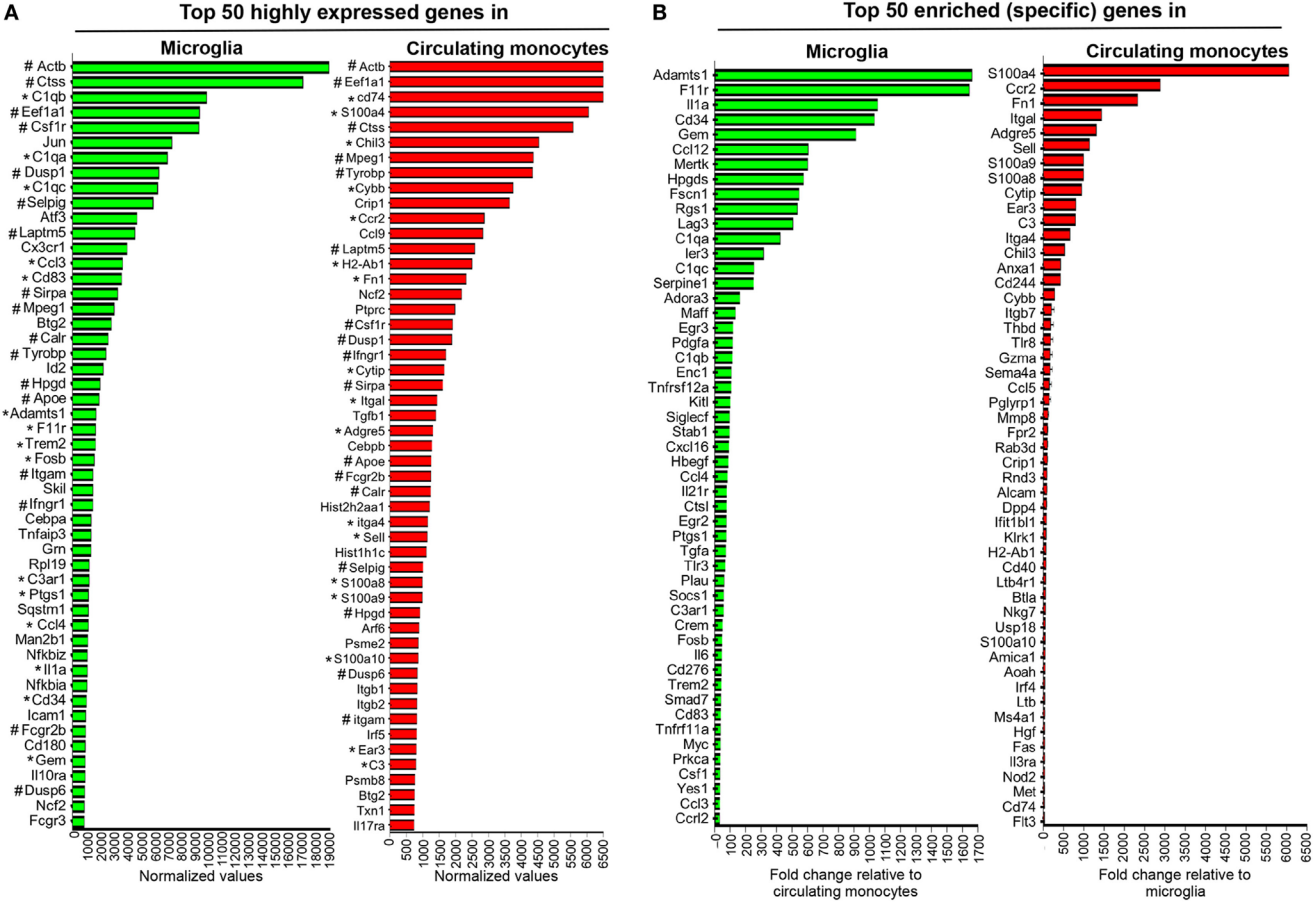
Gene expression characterizing microglia and circulating monocytes under homeostatic conditions. Spinal cord-derived microglia (CD45^low^CD11b^+^CX3CR1^GFP+^) and circulating blood monocytes (CD11b^+^CCR2^RFP+^) were purified from naïve CX3CR1^GFP/+^CCR2^RFP/+^ mice by FACS and RNA subjected to nCounter analysis using the myeloid cell probe panel. Panel **(A)** depicts the top 50 highly expressed genes and **(B)** the enriched genes uniquely characterizing each population. In **(A)** * highlights genes that are both highly expressed and enriched in each population, while # highlights genes highly expressed and common to both microglia and circulating monocytes.

As both microglia and infiltrating BMDM retained their phenotype throughout infection, CD45^low^CD11b^+^CX3CR1^GFPhi^CCR2^−^ and CD45^hi^CD11b^+^CX3CR1^GFPlow^CCR2^+^ populations were isolated by FACS from spinal cords at days 5, 7, 10, and 14 p.i. for subsequent mRNA expression analysis. Age-matched naïve animals were used to isolate microglia and blood circulating monocytes as precursors of CNS-infiltrating BMDM. Gene expression profiles for all purified populations were obtained using nCounter analysis and the Innate Myeloid Immune panel. The respective naïve populations were used to assess signature gene expression profiles under homeostatic conditions (Figure 3).

Figure 3A shows the top 50 highly expressed genes within each population relative to three nCounter platform housekeeping genes, namely G6pdx, Polr1b, and Tbp, selected for three high, medium, and low expression, respectively, in this part of analysis platform. Figure 3B lists the top 50 enriched genes specific for microglia compared to monocytes, or monocytes versus microglia, respectively. Among the top 50 genes highly expressed in microglia, 15 were also specific and included genes of the complement cascade (C1qa, C1qb, C1qc, and C3ar1) and Trem2, encoding a cell surface receptor involved in phagocytic functions and known to be expressed by microglia (39). Other genes, such as Adamts1, F11r or Hpgds, found within the top 50 enriched genes expressed by microglia (Figure 3B) were also previously described as microglia specific (17, 40). Cx3cr1 mRNA encoding the fractalkine receptor and used as a marker for microglia (41), was also among the top 50 highly expressed genes (Figure 3A), but not unique, consistent with the CX3CR1^lo^ phenotype on circulating monocytes. Similarly, CCR2 expression characteristic of monocytes was confirmed by ccr2 mRNA as the second in place of the top 50 expressed genes specific for circulating monocytes (Figure 3B). Other specific signature genes of monocytes are related to motility and migration/tissue invasion, e.g., S100a4, S100a8, S100a9, Fn1, Sema4, Mmp8, and to a lesser extent MHC class II antigen presentation, e.g., H2-Ab1, CD74, and Fas. Microglia and circulating monocytes also shared 17 highly expressed genes, including genes characteristic of the myeloid lineage such as Csf1r, a gene coding for a cell surface receptor essential for hematopoietic precursors differentiation into myeloid cells and Mpeg1, a gene coding for a membrane protein with a perforin domain expressed on myeloid cells (Figure 3A). Altogether, these results highlight unique as well as common basal expression signatures of each purified myeloid population, thus providing a basis for characterization of altered expression patterns following JHMV infection.

### BMDM and Microglia Display Overall Distinct Patterns of Gene Expression as Well as Temporal Regulation Throughout JHMV Infection

**Figure 4:**
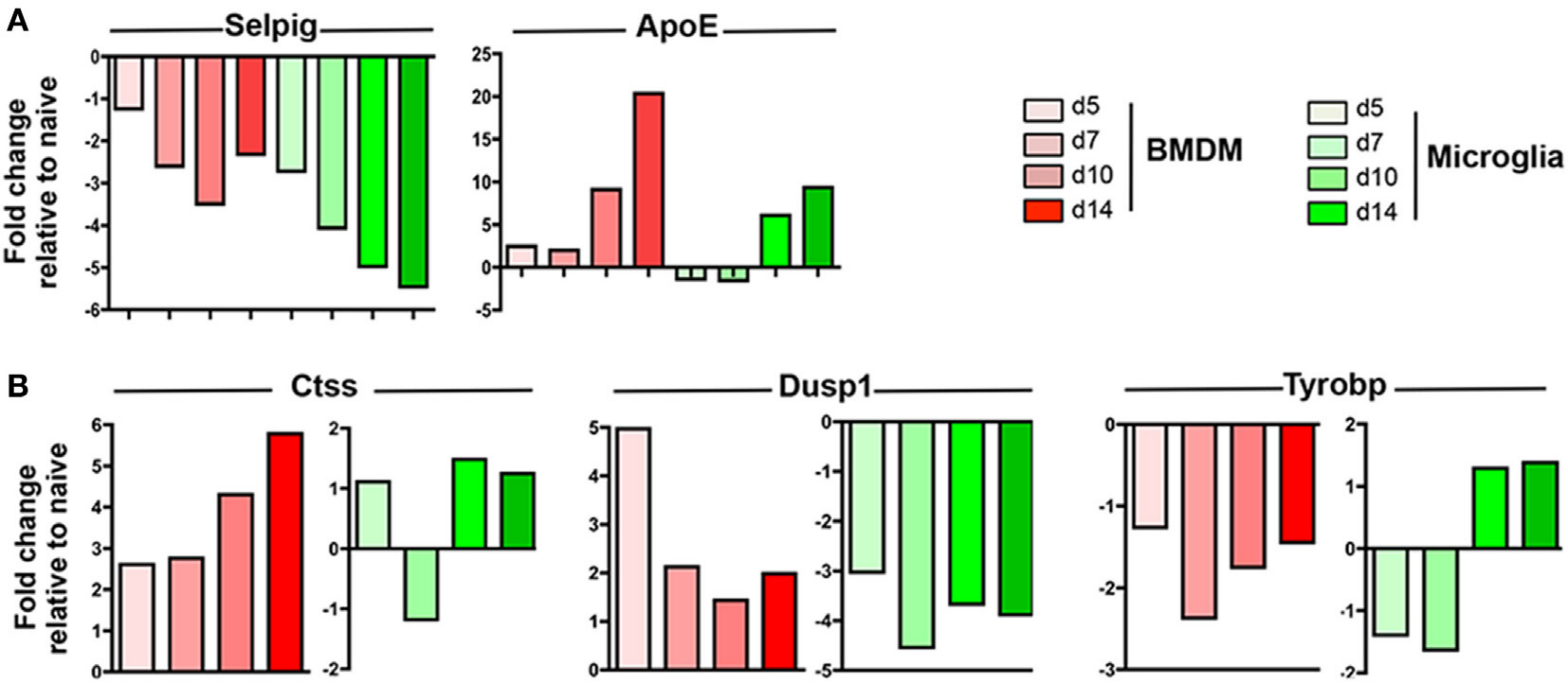
Expression profiles of genes commonly expressed by central nervous system (CNS) infiltrating bone marrow-derived macrophage (BMDM) and microglia during JHMV infection. FACS purified BMDM and microglia from spinal cords of JHMV-infected JHMV-infected CX3CR1^GFP/+^CCR2^RFP/+^ mice were assessed for differential gene expression by nCounter analysis using the myeloid cell probe panel. **(A)** Selpig and ApoE genes were chosen as representative genes showing similar expression patterns in BMDM and microglia **(B)** Ctss, Dusp1, and Tyrobp genes were chosen to highlight distinct regulation in BMDM and microglia throughout infection. Data are obtained from the spinal cords of seven-pooled mice for each time point and represent the fold increase relative to naïve levels at days 5, 7, 10, and 14 p.i. in BMDM and microglia.

Following JHMV infection, the majority of commonly expressed genes in microglia and circulating monocytes were regulated similarly within the CNS over time. For example, Selpig mRNA was downregulated at all time points during JHMV infection, while Apoe was specifically upregulated at days 10 and 14 p.i. in both BMDM and microglia (Figure 4A). Interestingly however, three genes among the common and highly expressed genes were regulated differently. Ctss mRNA, encoding for Cathepsin S, a lysosomal cysteine proteinase participating in the MHC Class II molecule antigen presentation pathway as well as nociception (42, 43), was specifically upregulated within BMDM, with highest levels observed at days 10 and 14 p.i. (Figure 4A), when demyelination increases. By contrast, microglia transiently downregulated Ctss mRNA at day 7 p.i. Opposite regulation was also observed for Dusp1 mRNA (Figure 4A). Dusp1 mRNA encodes the dual specificity protein phosphatase 1, an enzyme involved in the cellular stress response and a negative regulator of cell proliferation (44). While Dusp1 mRNA levels were vastly upregulated early following BMDM accumulation, but declined by day 7 p.i. and thereafter, levels were rapidly downregulated within microglia (Figure 4B). Finally, Tyrobp mRNA encoding for the TREM2 adaptor DAP12 and known to regulate microglia phagocytic functions (39), was downregulated in BMDM throughout infection, but specifically upregulated within microglia at days 10 and 14 p.i. (Figure 4B). This expression pattern on microglia correlated with the onset of myelin loss and supported TREM2 signaling specifically by microglia in response to tissue damage.

**Figure 5:**
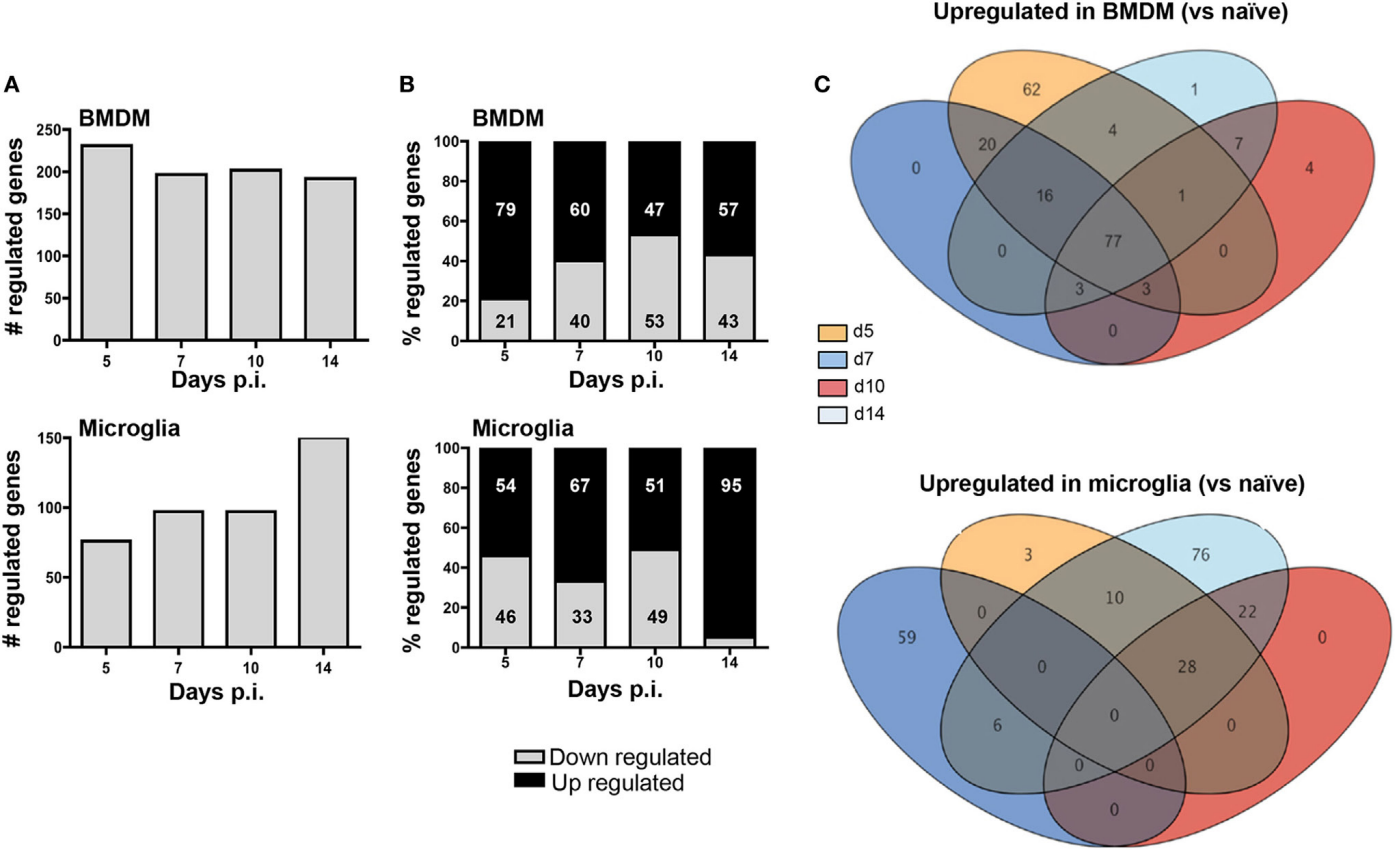
Infiltrating bone marrow-derived macrophage (BMDM) and microglia reveal distinct gene regulation following JHMV infection. BMDM and microglia gene expression patterns obtained from infected mice described in Figure 4 were analyzed for **(A)** the number of total regulated genes and **(B)** the relative distribution of up- and downregulated genes (percentage of increased and decreased) within each population. **(C)** The Venn diagram represents the number of genes upregulated at least twofold relative to the naïve populations throughout days 5, 7, 10, and 14 p.i. in BMDM and microglia. Numbers in overlapping ellipses represent genes upregulated at several time points.

To determine whether apparently differential functions of BMDM and microglia associated with JHMV-induced demyelination are reflected in distinct gene profiles, we monitored overall up- and downregulation of gene expression relative to basal levels in each population. Analysis times were chosen to correlate with innate responses (d5 p.i.), peak T cell effector function (d7 p.i.), resolution of infection and initiation of demyelination (d10 p.i.), and finally viral clearance and overt demyelination (d14 p.i.). At day 5 p.i. a higher number of genes were differentially regulated within infiltrating BMDM compared to microglia (231 versus 76; Figure 5A). Moreover, almost 80% of the genes showing altered expression in early infiltrated BMDM were increased compared to basal levels, while only 54% were increased in microglia; the remaining differentially expressed mRNAs were decreased (Figure 5B). The overall number of differentially expressed genes slightly declined in BMDM by day 7 p.i., when T cells exert maximal effector function (37), and remained fairly constant throughout day 14 p.i. (Figure 5A). Furthermore, the relative decline in the proportion of upregulated mRNAs coincided with an increased proportion of downregulated genes, reaching a roughly equal distribution at days 10–14 p.i., when virus is largely controlled (Figure 5B). In contrast, microglia altered their gene expression pattern extensively at day 7 p.i. (97 genes, Figure 5A) with 67% of differentially regulated genes showing increases (Figure 5B). By day 10 p.i., overall altered gene expression remained stable relative to day 7 p.i., with equal proportions showing increases and decreases. However, as myelin loss progresses by day 14 p.i., differentially regulated genes increased again in numbers, with the proportion of upregulated mRNAs reaching 95% (Figure 5A). Altogether these data show unique regulation of gene profiles in BMDM compared to microglia throughout the course of infection. While most changes were evident in BMDM following initial CNS accumulation, microglia revealed most pronounced changes at the time of myelin loss.

We further analyzed differential gene expression across time points focusing on upregulated genes using Venn diagrams to reveal the relative proportion of genes that were commonly increased at all time points (Figure 5C). Of the 183 upregulated genes in BMDM at day 5 p.i., 62 were unique to day 5. On the other hand, 77 genes (representing 42% of all upregulated genes) remained highly expressed at all other time points (Figure 5C). Of note, not a single gene transcript was specifically upregulated at day 7 p.i., and only three overlapped with sustained upregulation at days 10 and 14 p.i. Similarly, only 4 gene transcripts were specifically elevated at day 10 p.i., 7 were unique to both days 10 and 14, and only one was unique to day 14 p.i. These results indicate that the gene expression profile characterizing BMDM is established early following infection, with sparse unique alterations as BMDM decline during infection. In stark contrast, only 3 of 41 gene transcripts upregulated in microglia at day 5 p.i. were unique to day 5, and no gene transcript was commonly upregulated across all time points analyzed. Furthermore, distinct from BMDM, 59 gene transcripts were uniquely upregulated by day 7 p.i., with none common to day 10 p.i., and only six overlapping with those upregulated at day 14 p.i. Although no gene transcripts were upregulated uniquely at day 10 p.i., 22 overlapped with those upregulated at day 14 p.i. A further 76 genes, comprising 53% of all upregulated genes at day 14 p.i., were specifically expressed at elevated levels at day 14 p.i. coinciding with overt myelin loss (Figure 5C). These profiles reveal a dynamic range of responses and extensive plasticity of gene expression profiles in microglia throughout JHMV infection (Figure 5C).

### Gene Expression Profiles Characterizing BMDM and Microglia at Peak of Demyelination

**Figure 6:**
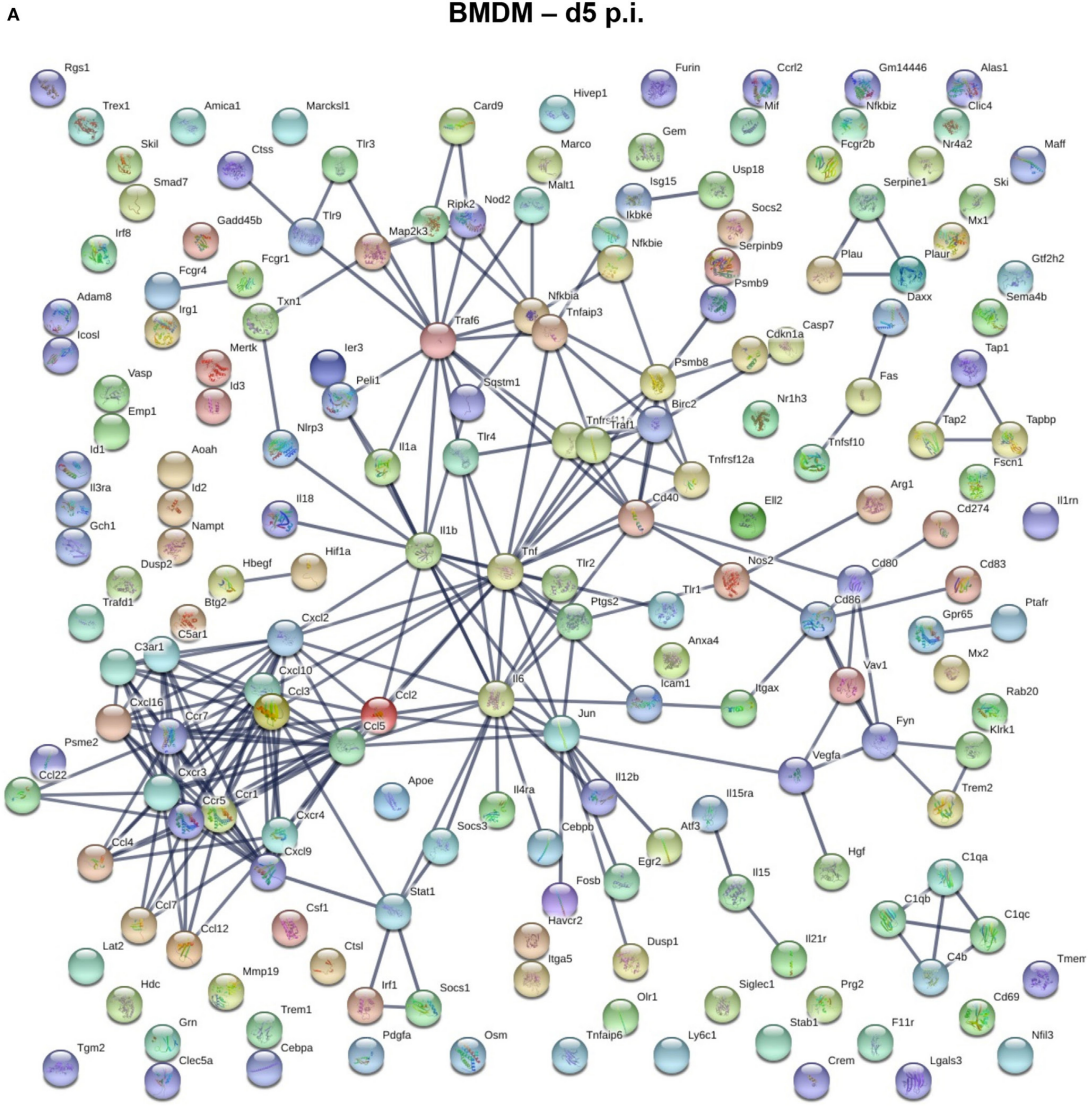

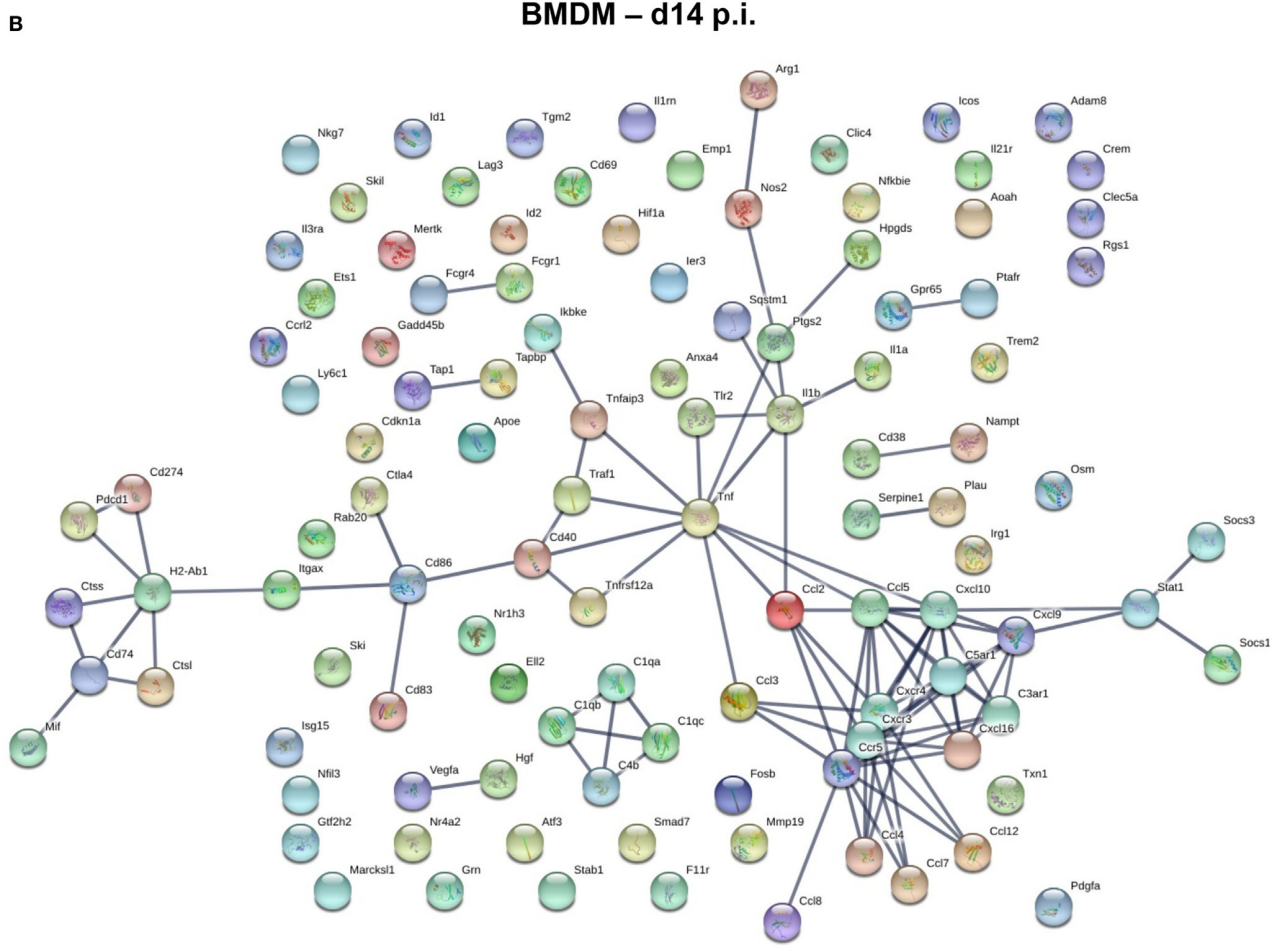
Upregulated gene network within central nervous system (CNS) infiltrating macrophages at d5 and d14 p.i. Protein–protein network constructed based on genes upregulated by at least twofold in infiltrated bone marrow-derived macrophage (BMDM) at days 5 **(A)** and 14 **(B)** p.i. compared to naïve levels were analyzed using Ingenuity IPA software.

**Figure 7:**
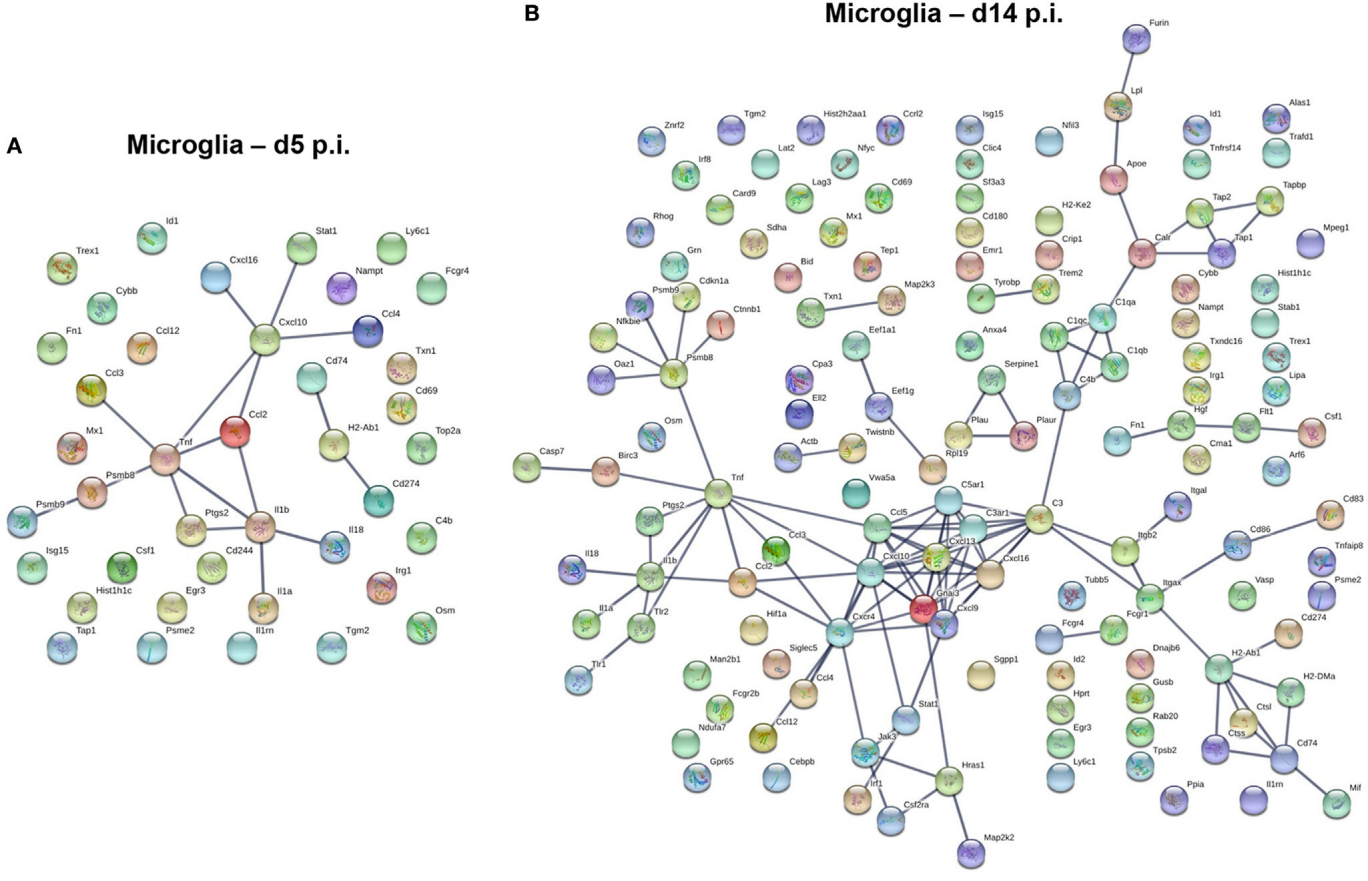
Upregulated gene network within microglia at d5 p.i. and peak demyelination. Protein–protein network constructed based on microglia genes upregulated by at least twofold at days 5 **(A)** and 14 **(B)** p.i. compared to naïve levels were analyzed using Ingenuity IPA software.

We next used a protein–protein network connection constructed based on differential gene expression to specifically examine upregulation of gene transcripts within BMDM and microglia correlating with demyelination at day 14 p.i. For comparison, we also analyzed the network connection at day 5 p.i., when expression profiles were most prominently altered in BMDM, but more modestly in microglia. This comparative analysis was chosen to provide clues about specific functions and involvement of microglia relative to BMDM in tissue destruction (Figures 6 and 7). Our initial focus was on temporally altered networks in BMDM (Figure 6). At day 5 p.i., early infiltrated BMDM expressed a wide array of chemokines regulating CNS infiltration of both innate (CXCL2, CCL2, CCL4, CCL5, CCL7, and CCL12) and adaptive (CXCL9, CXCL10) immune cells (Figure 6A). A large cluster of molecules regulating the innate immune response, essential to limit early viral replication (45, 46), was also expressed by BMDM (Figure 6A). These include pathogen recognition receptors such as TLRs (TLR1-4 and TLR9) and molecules linked to the TNF pathway (TNF, TRAF6, etc.). Finally, BMDM expressed molecules involved in antigen presentation, including Tap1 and Tap2, as well as T cell activating co-stimulatory molecules (CD80 and CD86) or IL-12, which induce Th1 differentiation (Figure 6A). These results indicate that early infiltrating BMDM orchestrate the acute innate immune response crucial for limiting CNS viral spread, as well as initiating the adaptive immune response by recruiting and activating T cells. At day 14 p.i., correlating with peak demyelination, a more restrained number of mRNA transcripts were upregulated in BMDM (Figure 6B). The cluster of chemokines mobilizing immune cells was sustained (Figure 6B). In contrast, the molecular network extended from TNF was more limited at d14 p.i. compared to d5 p.i. (Figures 6A,B). Molecules regulating primarily the CD4 T cell response were expressed at day 14 p.i. and comprised gene transcripts involved in antigen presentation including MHC class II (H2-Ab1) and co-stimulatory molecules such as CD80 and CD86 (Figure 6B). Interestingly, among the more restricted number of gene transcripts upregulated at day 14 p.i. in BMDM, several were transcripts encoding M2 molecules, which included Arg1, Il1rn and Tgm2 (Figure 6B).

In contrast to the vast number of genes upregulated early following infection in BMDM, a significantly lower number of upregulated genes characterized microglia at day 5 p.i. (Figure 7A). Transcripts for chemokines regulating migration of both innate and adaptive immune cells, such as CCL2, CCL3, CCL4, CCL12, CXCL10, and CXCL16, were expressed by microglia at day 5 p.i. (Figure 7A). Transcripts for TNF and other inflammatory cytokines generally associated with the innate responses, e.g., IL1a, IL1b, and IL18 were also upregulated early in microglia (Figure 7A). Another extended network of TNF comprised Psmb8 and Psmb9, subunits of the immuno-proteasome, essential for antigen presentation by MHC class I molecules. By day 14 p.i., the number of upregulated transcripts extensively increased in microglia (Figure 7B). The most clustered network comprised proteins like CCL5, CXCL9, CXCL10, CXCL13, CXCL16, and CXCR4, all chemokines and chemokine receptors regulating migration and arrest of adaptive immune cells within the CNS during inflammation (47). This chemokine cluster was linked to TNF and inflammatory cytokines previously detected at d5 p.i., e.g., IL1a, IL1b, and IL18. Another extended network of TNF comprised Psmb8 and Psm9, also present at d5 p.i., Ctnnb1 encoding b-catenin, a cellular adhesion molecule, and cdkn1a, a cyclin inhibitor. Other upregulated gene transcripts associated with class I antigen presentation, e.g., tap1 and tap2, were also linked through a network associated with complement component genes (C3, C3ar1, C4b, C1qa, C1qb, C1qc), which are highly expressed within microglia under homeostatic conditions (Figure 3). Similarly, tyrobp and Trem2, which formed phagocytic synapses (48), are both highly expressed in microglia during myelin loss. Finally, a wide variety of upregulated gene transcripts are associated with MHC class II antigen presentation and modulation of T cell function. This includes H2-Ab1, encoding for the MHC class II molecules and H2-DM, encoding for a second accessory protein, which facilitates peptide loading. Similarly, genes associated with the invariant chain of MHC class II were increasingly expressed within microglia, such as Cd74 and Ctss (Cathepsin S, which cleaves invariant chain thereby promoting loading on MHC Class II). In addition, genes encoding for modulators of the CD4 T cell response, such as Itgax (CD11c), Cd86 and Cd83 were also expressed by microglia (Figure 7B). Overall, the upregulated networks are related to complement activation, enhanced class I and class 2 antigen processing and presentation (potentially related to IFN-γ responses) as well as migration and phagocytic activity. However, there does not appear to be a bias toward phagocytic receptors over other components activated by pro-inflammatory mediators.

### Microglia Repressed Anti-Inflammatory Genes During JHMV-Induced Demyelination

**Figure 8:**
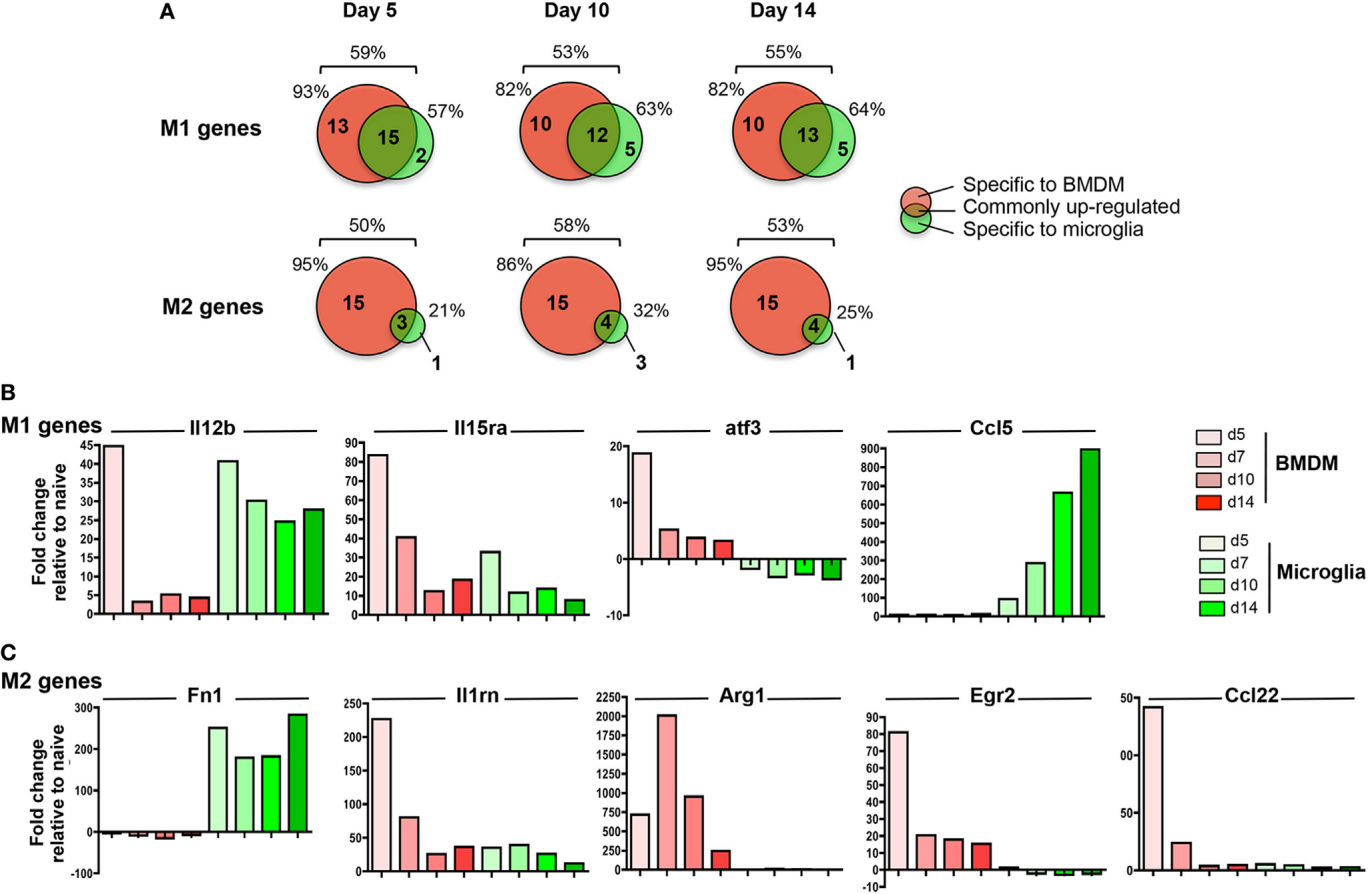
M1 and M2 gene regulation following JHMV infection. bone marrow-derived macrophage (BMDM) and microglia gene expression patterns obtained from infected mice described in Figure 4 were analyzed for **(A)** the number and distribution of M1 and M2 genes upregulated specifically in one or both populations at days 5, 10, and 14 p.i. Percentages above each diagram represent the overall proportion of M1 or M2 genes upregulated at each time point, while percentages on each side of the diagram represent the proportion of regulated genes with increased expression in BMDM versus microglia. The fold expression change of **(B)** select M1 genes (Il12b, Il15ra, atf3, Ccl5) and **(C)** select M2 genes (Fn1, Il1rn, Arg1, Egr2, Ccl22) compared to naïve levels within microglia and BMDM between days 5 and 14 p.i. is represented in bar graphs.

Pathogenic versus protective functions of myeloid cells following activation have also been correlated to expression of key molecules defined as M1 versus M2 markers. While the strict classification of myeloid cells into the M1 or M2 category has been tempered based on a more dynamic and mixed phenotype during inflammatory responses (49, 50), the M1 and M2 markers remain helpful to gage overall effector functions. Among the 89 analyzed gene transcripts in the Nanostring myeloid panel related to M1/M2 polarization (51 M1 and 38 M2 genes), between 50 and 59% were upregulated across the course of JHMV infection with no difference comparing M1 versus M2 genes (Figure 8A).

Among the total upregulated M1 markers, about 50% were commonly increased within both infiltrating BMDM and microglia; representative genes were Il12b and Il15Ra (Figures 8A,B). However, while high levels of Il12b mRNA were observed in both BMDM and microglia at d5 p.i., expression was only sustained in microglia at day 7 p.i. and thereafter (Figure 8B). By contrast, IL15ra was increased in both BMDM and to a lesser extent in microglia at d5 p.i., but was decreased in both populations at later time points p.i. (Figure 8B). In addition, between 35 and 45% of M1 markers were specifically expressed by infiltrating BMDM during the course of JHMV infection (Figure 8A), including Cd86, atf3, Ifng, Ptgs2, Ccr7, Cxcl11, and Cxcl12 transcripts (Figure 8B and data not shown). However, only 5 M1 related gene transcripts were specifically upregulated in microglia at the time of demyelination, including Ccl5, Fas, Cxcl13, Tnfsf10, and Psmb9. (Figures 8A,B and data not shown).

Importantly, the most prominent difference between microglia and BMDM was noted in M2 marker regulation. Among the 50–58% M2 gene transcripts upregulated following JHMV infection, only a small proportion (21–32%) was expressed by microglia (Figure 8A). Fn1 was the only M2 marker specifically expressed by microglia at the time of demyelination (Figure 8A). Although Il1rn transcript expression was elevated in both BMDM and microglia, the increase was at best modest in microglia (Figure 8C). The majority (86–95%) of M2 markers upregulated during JHMV infection were rather expressed by infiltrating BMDM, including Arg1, Erg2, Il-10, and Ccl22 (Figures 8A,C and data not shown). Increased transcript levels were most pronounces at days 5 and 7 p.i., but dropped off thereafter. Altogether, these data showed that while infiltrating BMDM express a mixed phenotype of M1 and M2 markers during JHMV infection, microglia expressed primarily pro-inflammatory genes while not expressing M2 markers.

## Discussion

Microglia and infiltrating macrophages are major components of MS active lesions (51). Their effector functions are highly heterogeneous as evidenced by both pathogenic and protective functions during the course of MS (7). They can promote tissue damage by releasing toxic and pro-inflammatory molecules, mediate demyelinated axons through phagocytosis as well as propagate inflammation by recruiting and activating adaptive immune cells. On the other hand, both populations also display protective functions by clearing myelin debris, which facilitates remyelination, as well as releasing trophic and anti-inflammatory factors, which promote tissue repair. While it remains a challenge to distinguish infiltrating macrophages from microglia in MS lesions due to morphological and phenotypic similarities, they are disparate effector cells based on animal MS models (17, 52). Questions relating to the pathogenicity of infiltrating macrophages and/or microglia in MS remain unanswered. Can both populations display protective functions? Do they display dynamic functions throughout the evolution of MS lesions? Deciphering the respective roles of macrophages versus microglia in facilitating tissue damage and/or repair is essential to our understanding of MS pathogenesis and development of effective therapeutic strategies.

In the murine EAE model of MS, infiltrating BMDM are essential in mediating demyelination (53). Gene expression profiles demonstrated that BMDM are indeed highly phagocytic and inflammatory at disease onset, while microglia display a repressed phenotype (17). By contrast, during JHMV-induced demyelination, recruited BMDM are dispensable for the demyelinating process (30). Distinct from EAE, where microglia activation precedes CNS infiltration of BMDM (52), JHMV infection elicits early BMDM infiltration, prior to microglia activation. These distinct kinetics of BMDM recruitment relative to microglia activation thus appear to correlate with the apparently opposing roles of microglia as demyelinating populations. These data further suggest that early responses set the stage or imprint subsequent effector functions of BMDM and microglia. Using a similar approach with Nanostring analysis as in EAE, the present study used gene expression profiling to characterize both BMDM and microglia myeloid functions at various times post JHMV infection. Analysis of overall gene expression patterns revealed that the most extensive changes in BMDM were evident early after infection, while microglia showed a more dynamic profile throughout the course of viral encephalomyelitis. Importantly, the most drastic gene upregulation in microglia was observed coincident with demyelination, at which time peak viral load and T cell effector function have substantially subsided (54). Our data contrast with EAE (17), where BMDM upregulated far more genes compared to microglia at disease onset, supporting opposing functions of BMDM and microglia in mediating demyelination in these two models. Further, while BMDM exhibited a mixed expression profile of both pro- and anti-inflammatory markers, microglia expressed a highly pro-inflammatory profile and repressed most of the M2 markers across the entire time course of JHMV encephalomyelitis.

Analysis of protein-interacting networks within genes upregulated in microglia at the time of myelin loss revealed several key functions linked to promoting tissue damage. Genes associated with complement activation were notably increased, although they were already highly expressed by microglia under homeostatic conditions. Complement activation as a pathogenic component in MS has been reported following detection of deposits of the activated products of the complement component C3 in MS lesions (55). The classical complement pathway has also been shown to mediate OLG death thus promoting demyelination (56). Microglia phagocytic activity may also initiate tissue damage by directly removing myelin from axons, especially at the node of Ranvier (17). Genes associated with TREM2/DAP12 signaling were also highly expressed by microglia at time of demyelination. TREM2 modulates phagocytic capacity of myeloid cells *via* DAP12 signaling (57) and is expressed on myelin-loaded myeloid cells in MS lesions (58), supporting a role in MS pathogenesis. Similar to JHMV infection, TREM2 is predominantly expressed by microglia during EAE and cuprizone-induced demyelination (59–61). However, TREM2-modulated phagocytic functions are essential for removal of myelin debris and remyelination implicating repair-promoting functions of microglia in the specific tissue environments defining these two demyelination models (17, 62). Preferential TREM2 expression within microglia compared to BMDM following JHMV infection support a more pathogenic role of TREM-2 in JHMV-induced demyelination, potentially by promoting myelin stripping after recognition of glycolipids and phospholipids exposed on damaged myelin. In this context, it is critical to note that JHMV infection is associated with extensive transient production of IFN-γ and its inducible genes, i.e., iNOS, and CXCR3 ligands, which may drive a more phagocytic pathway in microglia in efforts to remove damaged proteins and lipids (54). Further investigation is required to define inflammatory conditions under which TREM-2 modulated or other phagocytic pathways promote tissue damage or repair and whether these are transient and reversible. Microglia may also induce demyelination by secreting toxic factors including inflammatory cytokines that are highly expressed by microglia at time of demyelination, including TNF. TNF can induce OLG death (63, 64) and is expressed in active MS lesions, as well as elevated TNF in serum and cerebral spinal fluid correlates with enhanced MS pathology (65, 66). Finally, microglia functions during JHMV infection were also associated with promoting adaptive immune response. An extensive network of chemokines and chemokines receptors relating to the recruitment and arrest of T and B cells within the inflamed CNS were highly expressed by microglia. Similarly, several genes associated with antigen processing and presentation by MHC class I and II molecules were upregulated within microglia, suggesting that microglia promote T cell reactivation upon CNS entry. However, microglia explanted during EAE, TMEV as well as JHMV infections failed to support myelin-specific CD4 T cell responses *ex vivo*, despite detection of internalized myelin (67–69). A potential deficit in antigen processing was supported by the ability of exogenous peptide to overcome the inability of microglia to prime myelin-specific CD4 T cells (69). Nevertheless, this notion is opposed by our microglia profiling showing upregulation of genes involved in protein degradation and class II peptide loading, e.g. CD74 (Invariant chain), H2-DMa (peptide loading), Ctss (Cathepsin S), which cleaves invariant chain. The apparent inability of microglia to elicit CD4 T cell effector function *ex vivo* thus remains intriguing.

Our present study reveals new insights into the plasticity of microglia in adapting to inflammation and expressing pathogenic functions associated with demyelination, characteristics which have previously been ascribed to BMDM (17). Moreover, altered BMDM expression profiles coincided with their early infiltration into the CNS and remained largely similar throughout infection. While altered microglia gene expression coincided with the time of early, yet robust demyelination, it remains to be determined whether these changes are sustained at later time points during JHMV persistence, when clinical disease improves and remyelination occurs. It will be of specific interest to assess whether the microglia pro-inflammatory phenotype evolves to an anti-inflammatory, repair-promoting phenotype, as evidenced by the plasticity of myeloid cells in CNS autoimmunity (70). Furthermore, our study emphasizes that the distinct tissue environments during EAE and JHMV infection drive opposite effector functions of microglia versus infiltrating macrophages. The interplay between T cells, infiltrating macrophages and microglia, as well as astrocytes drives MS pathogenesis, yet mechanisms ultimately leading to loss of repair remain unclear. Taking advantage of demyelinating models characterized by distinct inflammatory factors such as both Th1 and Th17 cells in EAE (11), strong Th1 polarized responses during JHMV infection, distinct kinetics of BMDM recruitment versus glia activation promises to reveal essential new insights into the interplay of microglia and BMDM functions in debris clearance versus active myelin stripping and ongoing axonal damage. Longitudinal studies will aid in developing efficient future therapies to combat MS pathogenesis.

## Ethics Statement

All procedures were performed in compliance with protocols approved by the Cleveland Clinic Institutional Animal Care and Use Committee.

## Author Contributions

CS designed and performed experiments, collected and interpreted the data, and wrote the manuscript. RD analyzed data and edited manuscript. CB designed the research, provided materials, interpreted the data, and wrote the manuscript. All authors approved the final manuscript.

## Conflict of Interest Statement

The authors declare that the research was conducted in the absence of any commercial or financial relationships that could be construed as a potential conflict of interest.

## Supplementary Material

Figure S1 Validation of Nanostring nCounter gene expression analysis by real-time PCR. To validate Nanostring nCounter data, expression of IL-15, Il1Rn, and Arg1 was analyzed by Q-PCR in naïve circulating monocytes and microglia, as well as in bone marrow-derived macrophage (BMDM) and microglia isolated from the spinal cord of JHMV-infected mice at days 5, 7, 10, and 14 p.i. Levels of mRNA expression are normalized to GAPDH mRNA levels.

## Abbreviations

BMDM, bone marrow-derived macrophages; CNS, central nervous system; EAE, experimental autoimmune encephalitis; MHC, major histocompatibility complex; mAb, monoclonal antibody; MHV, mouse hepatitis virus; MS, multiple sclerosis; NO, nitric oxide; ROS, reactive oxygen species; OLG, oligodendrocytes; TMEV, Theiler’s murine encephalomyelitis virus; TNF, tumor necrosis factor; WT, wild-type.
