# Supplementary figures and images for: Distinct Gene Profiles of Bone Marrow-Derived Macrophages and Microglia During Neurotropic Coronavirus-Induced Demyelination
Source: Front Immunol. 2018 Jun 11;9:1325. doi: 10.3389/fimmu.2018.01325 (PMC6004766; doi:10.3389/fimmu.2018.01325)

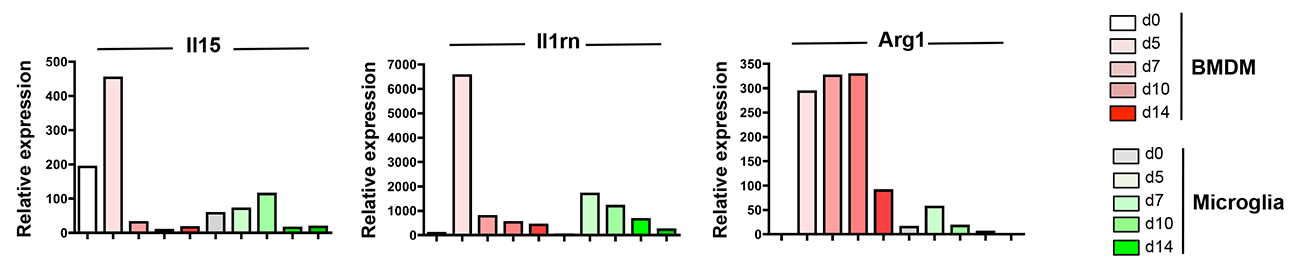

Supplement: Figure S1 — Validation of Nanostring nCounter gene expression analysis by real-time PCR. To validate Nanostring nCounter data, expression of IL-15, Il1Rn, and Arg1 was analyzed by Q-PCR in naïve circulating monocytes and microglia, as well as in bone marrow-derived macrophage (BMDM) and microglia isolated from the spinal cord of JHMV-infected mice at days 5, 7, 10, and 14 p.i. Levels of mRNA expression are normalized to GAPDH mRNA levels. [file image_1.TIF]
